# Ultra-low 70-kV cardiac CT angiography in neonates: a comparative dose-optimized approach with preserved image quality

**DOI:** 10.3389/fcvm.2026.1882665

**Published:** 2026-08-19

**Authors:** Xiaofang He, Chi Nie, Xiaoyan Zheng, Lei Zheng, Chenxiao Wang, Ruqi Fang, Yumin Zhong

**Affiliations:** 1 Department of Radiology, Fujian Children's Hospital (Fujian Branch of Shanghai Children's Medical Center), College of Clinical Medicine for Obstetrics & Gynecology and Pediatrics, Fujian Medical University, Fuzhou, China; 2 Diagnostic Imaging Center, Shanghai Children's Medical Center, Shanghai Jiao Tong University School of Medicine, Shanghai, China

**Keywords:** cardiac angiography, cardiac CT, congenital heart disease, iodine delivery, low tube voltage, neonate, radiation dose

## Abstract

**Objective:**

To evaluate the feasibility of ultra–low tube voltage (70 kV) cardiac CT angiography (CTA) with weight-adapted iodine delivery in neonates and to compare image quality, coronary visibility, radiation exposure, and iodine delivery with those of a conventional 80-kV protocol.

**Methods:**

This retrospective study included 125 neonates with congenital heart disease who underwent prospectively ECG-triggered cardiac CTA using a 256-row CT scanner. Patients were divided into an 80-kV group reconstructed with 50% adaptive statistical iterative reconstruction-V (ASIR-V) (*n* = 61) and a 70-kV group reconstructed with 70% ASIR-V (*n* = 64). Both protocols used standardized weight-adapted iodine delivery (450 mg I/kg). Subjective and objective image quality, coronary artery visibility, radiation dose, and iodine delivery were compared between groups.

**Results:**

Overall interobserver agreement for subjective image quality assessment was excellent (*κ* = 0.815). Subjective image quality scores were comparable between the two groups across most coronary segments, with no significant differences in overall image quality (3.98 ± 1.04 vs. 3.95 ± 1.05, *P* = 0.545). Objective image quality parameters, including CT attenuation, image noise, signal-to-noise ratio, and contrast-to-noise ratio, also showed no significant differences between groups (all *P* > 0.05). Coronary artery visibility remained high in both groups, with overall visibility rates exceeding 89%. Importantly, distal coronary segments remained diagnostically assessable in approximately 75% of cases under the 70-kV protocol. Compared with the 80-kV protocol, the 70-kV protocol achieved a 24% reduction in effective radiation dose (0.32 ± 0.05 vs. 0.42 ± 0.06 mSv, *P* < 0.001) without increasing iodine delivery.

**Conclusions:**

Ultra-low tube voltage (70 kV) cardiac CT with weight-adjusted iodine delivery is feasible for neonatal imaging and can substantially reduce radiation exposure while preserving coronary artery image quality and visibility. This approach may help achieve a more balanced trade-off among image quality, radiation dose, and contrast administration in neonatal cardiac CT examinations.

## 1 Introduction

Congenital heart disease (CHD) is frequently associated with coronary artery anomalies, which have a reported prevalence of approximately 8.16% in children (1). Accurate delineation of the origin and course of the coronary arteries is critical for surgical planning in complex CHD (2, 3). With advances in multidetector CT technology, cardiac CT angiography has emerged as an important noninvasive imaging modality for evaluating coronary anatomy in pediatric patients (4).

However, accumulating epidemiological evidence suggests a potential association between CT exposure in childhood and an increased long-term risk of malignancy (5). In addition, immature renal function and limited circulating blood volume in neonates necessitate careful management of iodine delivery (6). Consequently, minimizing radiation exposure and iodine delivery while maintaining adequate coronary visualization has become a central focus in pediatric cardiac imaging (6–8).

Lowering tube voltage is a well-established strategy for reducing radiation exposure while preserving vascular enhancement (9–11). However, further reduction in tube voltage may increase image noise and potentially compromise diagnostic image quality. Owing to their small body size and shorter x-ray penetration paths, neonates may particularly benefit from ultra-low tube voltage imaging. Nevertheless, most published pediatric cardiac CT studies have employed 80-kV protocols, and evidence supporting the feasibility of 70-kV imaging in neonates remains limited. Therefore, whether a 70-kV protocol can further optimize radiation dose while maintaining adequate coronary image quality in this population warrants investigation (7, 12, 13).

Therefore, this study aimed to evaluate the feasibility of 70-kV cardiac angiography with a reduced, weight-adapted iodine delivery (450 mg I/kg) in neonates. Image quality, coronary artery visibility, radiation dose, and iodine delivery were systematically assessed to determine whether adequate coronary visualization can be preserved despite further reductions in radiation exposure.

## 2 Methods and materials

### 2.1 Study design and patients

**Figure 1:**
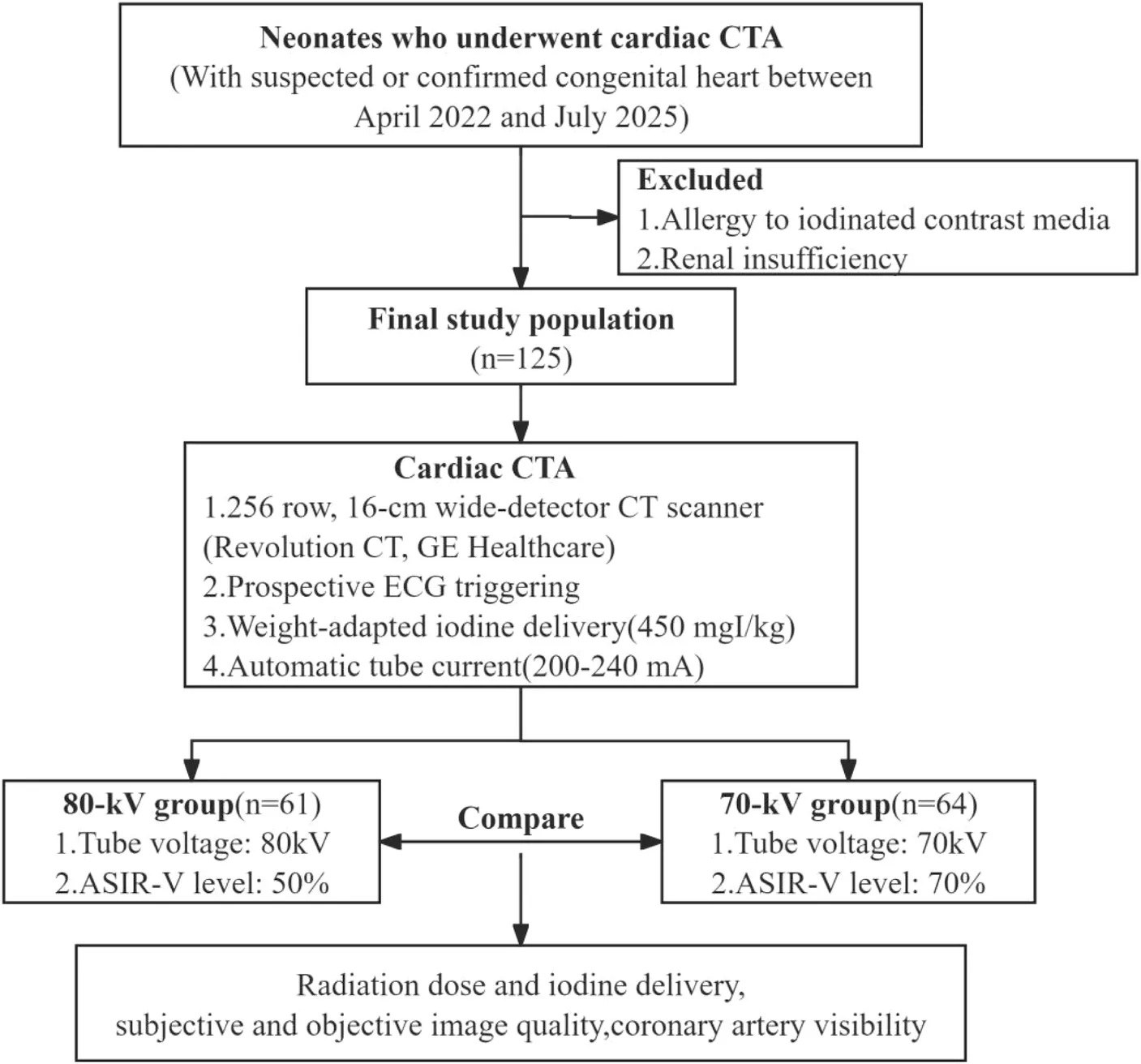
Flowchart of study design. ASIR-V, adaptive statistical iterative reconstruction.

This retrospective cohort study was approved by the Institutional Review Board of Fujian Children's Hospital (approval number: 2025ETKLRK10002). Written informed consent was obtained from the parents or legal guardians of all participants. Consecutive neonates with suspected or confirmed congenital heart disease who underwent contrast-enhanced cardiac CT before cardiac catheterization or surgical intervention between April 2022 and July 2025 were included. Exclusion criteria included allergy to iodinated contrast media and renal insufficiency. A total of 125 neonates were included in the study. Patients were categorized according to the routine clinical imaging protocol implemented during the study period. Before introduction of the optimized scanning protocol, cardiac CT examinations were routinely performed using an 80-kV protocol reconstructed with 50% ASIR-V blending (*n* = 61). After the optimized scanning protocol was introduced into routine clinical practice, consecutive patients underwent cardiac CT using a 70-kV protocol reconstructed with 70% ASIR-V blending (*n* = 64). CT findings were confirmed by cardiac catheterization and/or surgical findings (Figure 1).

### 2.2 Imaging examination

All examinations were performed under sedation, with oral chloral hydrate administered at a dose of 0.5 mL/kg before scanning. Cardiac CT angiography was performed using a 256-row, 16-cm wide-detector CT scanner (Revolution CT, GE Healthcare) in a prospectively ECG-triggered axial scanning mode. Tube current was automatically modulated within a range of 200–240 mA. Because all neonates had heart rates above 80 beats/min during scanning, the prospective ECG-triggered acquisition window was centered at 40%–50% of the R-R interval. Patients were positioned supine and scanned in a craniocaudal direction, with the scan range extending from the thoracic inlet to the level of the diaphragm. Continuous ECG monitoring was performed throughout the examination. Contrast medium (Iopromide, 300 mg I/mL, Bayer Healthcare) was administered via an antecubital vein using a dual-head power injector (Salient, Bayer Healthcare) at 1.5 mL/kg and an injection rate of 1–1.5 mL/s, corresponding to an iodine delivery of 450 mg I/kg. An equal volume of saline was subsequently injected at the same rate. Bolus tracking was performed with the region of interest placed in the left atrium at the four-chamber level. Scanning was automatically initiated when the attenuation reached the predefined threshold of 160 HU using the minimum scanner delay. No additional post-threshold delay was applied, and the same contrast-triggering protocol was used for all patients regardless of the underlying congenital cardiac pathology. The trigger threshold of 160 HU was selected according to our institutional neonatal cardiac CT angiography protocol. The application of bolus-tracking techniques for contrast timing optimization has been reported in previous pediatric cardiac CT angiography studies (14). Heart rate at the time of scanning was recorded. Image reconstruction was performed using ASIR-V combined with a cardiac motion correction algorithm (Snapshot Freeze, SSF). Images were reconstructed with a slice thickness and interval of 0.625 mm at 5% increments within the predefined systolic acquisition window. Depending on the individual cardiac motion characteristics, multiple cardiac reconstruction phases were generated for each examination. The reconstruction phase demonstrating the optimal coronary artery image quality was selected for subsequent subjective and objective image quality assessment. Different ASIR-V blending levels were applied according to the routine clinical imaging protocol: 50% ASIR-V for the 80-kV group and 70% ASIR-V for the 70-kV group. The selected reconstruction settings reflected routine institutional clinical practice during the study period and were not modified for the purposes of this retrospective study.

### 2.3 Image post-processing and analysis

Objective and subjective image quality were evaluated. All datasets were transferred to a dedicated workstation (AW 4.7, GE Healthcare) for post-processing, including multiplanar reformation (MPR), curved planar reformation (CPR), and maximum intensity projection (MIP). Subjective image quality was independently evaluated by two radiologists specialized in cardiovascular imaging with more than 5 years of experience in pediatric cardiac imaging, who were blinded to imaging protocols and clinical information. The coronary arteries were segmented into 11 standard segments: the origins of the left and right coronary arteries (LCAo, RCAo), left main coronary artery (LM), proximal, middle, and distal right coronary artery (RCAp, RCAm, RCAd), proximal, middle, and distal left anterior descending artery (LADp, LADm, LADd), and proximal and distal left circumflex artery (LCXp, LCXd).Each segment was scored using a five-point Likert scale (5, excellent without artifacts; 4, minor artifacts with high diagnostic confidence; 3, moderate blurring artifact with acceptable diagnostic quality; 2, poor visualization with limited diagnostic value; 1, non-diagnostic). In cases of disagreement, the lower score was assigned. Segments with scores ≥3 were considered diagnostically acceptable, and coronary segment visibility was defined as the proportion of evaluable segments. Objective image quality was assessed by a cardiovascular radiologist with more than 5 years of experience in pediatric cardiac imaging. Image noise was defined as the standard deviation (SD) of CT attenuation measured in subcutaneous thoracic fat at the level of the aortic root. Circular regions of interest (ROIs) were placed in the proximal segments of the RCA, LAD, and LCX to measure vessel attenuation and its SD. Signal-to-noise ratio (SNR) was calculated as vessel attenuation divided by its SD, and contrast-to-noise ratio (CNR) as the difference between vessel and fat attenuation divided by the SD of fat, according to previously described methods (15). ROI size and placement were maintained consistent across groups and positioned away from vessel walls and artifacts to minimize measurement bias.

### 2.4 Radiation and contrast dose assessment

Volume CT dose index (CTDIvol) and dose–length product (DLP) were recorded for each examination. Effective dose (ED) was estimated by multiplying the DLP by a neonatal-specific conversion coefficient of 0.039 mSv/(mGy·cm), as previously applied in pediatric cardiac CT studies; the estimation followed the dosimetric framework and tissue-weighting recommendations of ICRP Publication 103 (16, 17). Total iodine delivery was recorded as the administered iodine mass and expressed in grams of iodine (g I).

### 2.5 Statistical analysis

Statistical analyses were performed using SPSS software (version 22.0; IBM Corp, Armonk, NY, USA). Continuous variables were tested for normality using the Shapiro–Wilk test. Normally distributed data were presented as mean ± standard deviation and compared between groups using the independent-samples *t* test. Non-normally distributed data were expressed as median with interquartile range and compared using the Mann–Whitney *U* test. Categorical variables were presented as counts (percentages) and compared using the chi-square test. Interobserver agreement for subjective image quality was assessed using Cohen's kappa statistic. Kappa values were interpreted as follows: 0.41–0.60, moderate agreement; 0.61–0.80, good agreement; and >0.80, excellent agreement. All statistical tests were two-sided, and a *P* value < 0.05 was considered statistically significant.

## 3 Results

### 3.1 Clinical characteristics

A total of 125 neonates were included in this study. The most common diagnoses were ventricular septal defect (*n* = 34), patent ductus arteriosus (*n* = 24), coarctation of the aorta (*n* = 21), and total anomalous pulmonary venous connection (*n* = 9). Other congenital heart diseases included pulmonary artery stenosis (*n* = 7), complete transposition of the great arteries (*n* = 4), double-outlet right ventricle (*n* = 4), atrial septal defect (*n* = 3), pulmonary atresia with or without ventricular septal defect (*n* = 6), interrupted aortic arch (*n* = 3), tetralogy of Fallot (*n* = 2), double aortic arch (*n* = 2), cortriatriatum (*n* = 2), mitral atresia (*n* = 1), atrioventricular septal defect (*n* = 1), coronary artery fistula (*n* = 1), and right-sided aortic arch with aberrant left subclavian artery (*n* = 1). Congenital coronary artery anomalies (CAAs) were identified in four patients. Three cases were observed in the 80-kV group, including two coronary artery fistulas and one anomalous origin of the left coronary artery from the pulmonary artery. One case in the 70-kV group was identified as an anomalous origin of the right coronary artery from the left coronary sinus. All CAAs were surgically confirmed.

The mean age was 14.6 ± 10.0 days (range, 1–30 days), the mean body weight was 3.0 ± 0.8 kg (range, 1.3–5.5 kg), and the mean heart rate was 130.6 ± 16.7 beats/min (range, 100–187 beats/min). Baseline characteristics were generally comparable between the two groups, with no significant differences in age, body weight, or heart rate (all *P* > 0.05). However, sex distribution differed significantly between groups (*P* = 0.016). Detailed baseline characteristics are summarized in Table 1.

**Table 1:** Patient characteristics.

Groups	No. of patients	Male sex, *n* (%)	Age (days)	Weight (kg)	Heart rate (bpm)
80-kV group	61	34 (55.7%)	15.5 ± 10.3	3.0 ± 0.9	128.8 ± 17.8
70-kV group	64	42 (65.6%)	13.8 ± 9.6	3.1 ± 0.7	132.4 ± 15.6
*P* value	-	0.016	0.322	0.752	0.222
In total	125	76 (60.8%)	14.6 ± 10.0	3.0 ± 0.8	130.6 ± 16.7

### 3.2 Comparison of the subjective evaluation of image quality

Among all 1,375 coronary artery segments, overall interobserver agreement was excellent (*κ* = 0.815). The 80-kV group demonstrated slightly higher interobserver agreement than the 70-kV group (*κ* = 0.857 vs. *κ* = 0.774).

Segment-based analysis revealed variability in interobserver agreement across different coronary segments. In both groups, the coronary ostia and left main coronary artery demonstrated excellent agreement (*κ* > 0.80). Good-to-excellent agreement was also observed for the proximal, middle, and distal coronary segments in both groups. Numerically higher *κ* values were observed in the 80-kV group across most coronary segments. As *κ* statistics primarily represent descriptive measures of agreement, no formal statistical comparison between groups was performed. Detailed segment-based *κ* values are summarized in Table 2. All *κ* values were statistically significant (all *P* < 0.001).

**Table 2:** Interobserver agreement for subjective image quality assessment of coronary artery segments in the 80-kV and 70-kV groups.

Segment	80-kV group (*n* = 61)	70-kV group (*n* = 64)
Ostial segments	0.896	0.898
LCAo	0.914	0.920
RCAo	0.882	0.876
Proximal segments	0.884	0.785
LM	0.902	0.923
LADp	0.846	0.745
LCXp	0.845	0.755
RCAp	0.912	0.723
Middle segments	0.824	0.698
LADm	0.834	0.756
RCAm	0.809	0.635
Distal segments	0.762	0.684
LADd	0.772	0.702
LCXd	0.840	0.653
RCAd	0.706	0.682
All segments	0.857	0.774

Data are presented as Cohen's *κ* coefficients. *κ* > 0.80 indicates excellent agreement, and *κ* = 0.61–0.80 indicates good agreement. All *κ* values were statistically significant (*P* < 0.001). No formal statistical comparison between groups was performed. LCAo, left coronary artery ostium; RCAo, right coronary artery ostium; LM, left main coronary artery; LADp, proximal left anterior descending artery; LADm, middle left anterior descending artery; LADd, distal left anterior descending artery; LCXp, proximal left circumflex artery; LCXd, distal left circumflex artery; RCAp, proximal right coronary artery; RCAm, middle right coronary artery; RCAd, distal right coronary artery.

Subjective image quality scores for each coronary artery segment are summarized in Table 3. Overall image quality remained high in both groups, with no significant difference between the 80-kV and 70-kV groups (3.98 ± 1.04 vs. 3.95 ± 1.05, *P* = 0.545). Segment-based analysis demonstrated comparable image quality between the two protocols for proximal, middle, and distal coronary segments (all *P* > 0.05). Notably, the 80-kV group showed slightly higher scores in the coronary ostial segments (4.84 ± 0.39 vs. 4.70 ± 0.56, *P* = 0.019), particularly at the left coronary artery ostium (4.89 ± 0.32 vs. 4.70 ± 0.53, *P* = 0.029). However, both groups achieved near-maximal scores at these locations. No statistically significant differences in subjective image quality scores were observed for the remaining coronary segments (all *P* > 0.05).

**Table 3:** Comparison of subjective image quality scores between the 80-kV and 70-kV groups.

Segment	All patients (*n* = 125)	80-kV group (*n* = 61)	70-kV group (*n* = 64)	*P* value
Ostial segments	4.77 ± 0.49	4.84 ± 0.39	4.70 ± 0.56	0.019
LCAo	4.79 ± 0.45	4.89 ± 0.32	4.70 ± 0.53	0.029
RCAo	4.74 ± 0.52	4.80 ± 0.44	4.69 ± 0.59	0.248
Proximal segments	4.32 ± 0.81	4.31 ± 0.82	4.33 ± 0.81	0.700
LM	4.61 ± 0.63	4.52 ± 0.72	4.69 ± 0.53	0.180
LADp	4.42 ± 0.80	4.43 ± 0.85	4.41 ± 0.75	0.664
LCXp	3.87 ± 0.87	3.87 ± 0.81	3.87 ± 0.93	0.914
RCAp	4.38 ± 0.76	4.41 ± 0.76	4.36 ± 0.76	0.658
Middle segments	3.74 ± 0.97	3.75 ± 0.95	3.72 ± 0.99	0.748
LADm	3.88 ± 0.98	3.87 ± 0.97	3.89 ± 0.99	0.938
RCAm	3.59 ± 0.93	3.64 ± 0.91	3.55 ± 0.96	0.622
Distal segments	3.10 ± 0.99	3.12 ± 0.99	3.09 ± 0.99	0.915
LADd	3.08 ± 0.99	3.10 ± 1.01	3.06 ± 0.97	0.856
LCXd	2.86 ± 0.96	2.89 ± 0.99	2.83 ± 0.94	0.911
RCAd	3.38 ± 0.96	3.38 ± 0.93	3.38 ± 1.00	0.794
All segments	3.96 ± 1.05	3.98 ± 1.04	3.95 ± 1.05	0.545

Data are presented as mean ± standard deviation. Comparisons between groups were performed using the Mann–Whitney *U* test.

**Figure 2:**
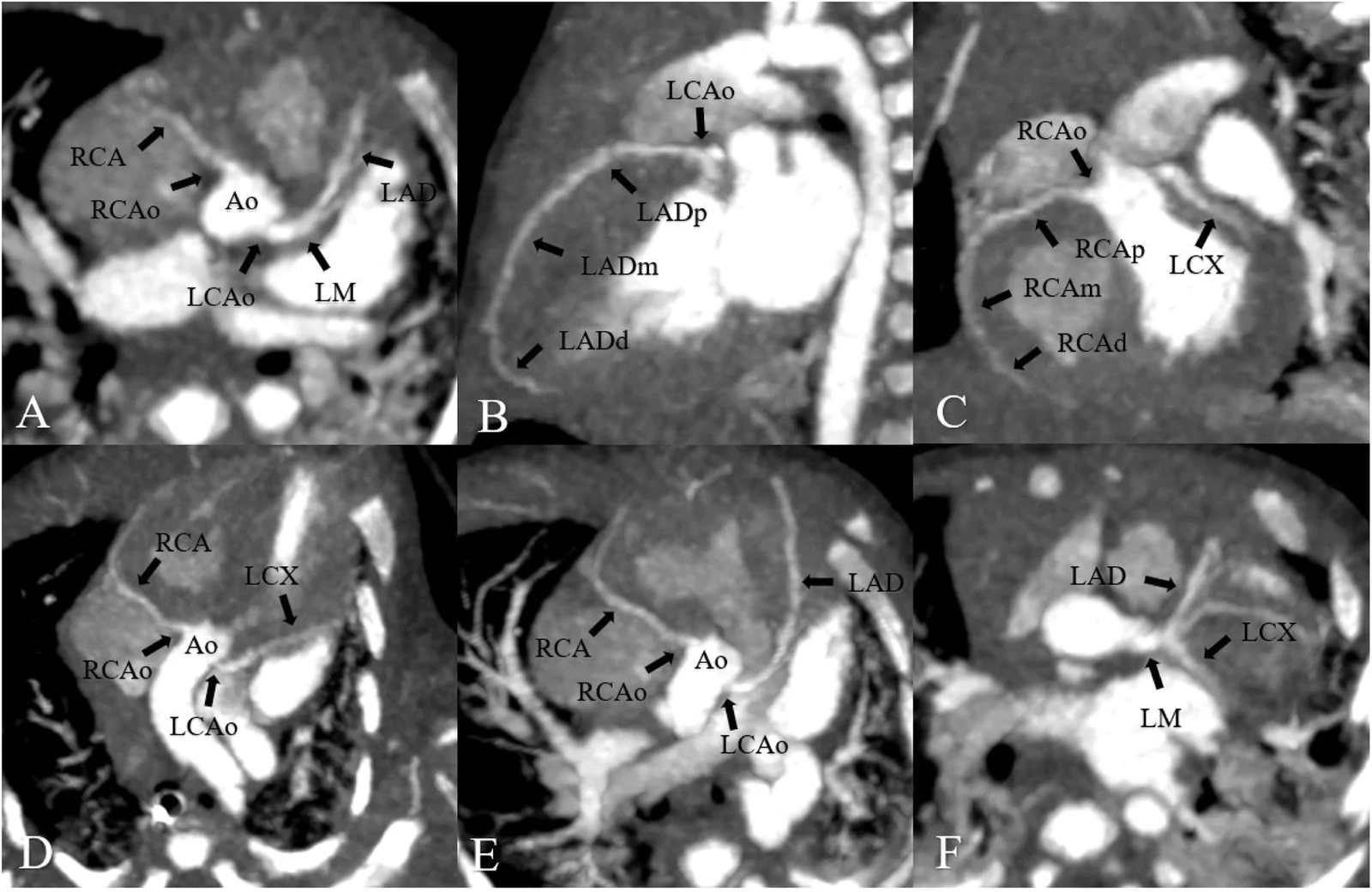
Representative coronary CT angiography images acquired using the 70-kV protocol in a neonate with ventricular septal defect. Multiplanar reconstruction (MPR), curved planar reformation (CPR), and maximum intensity projection (MIP) images demonstrate the visualization of coronary artery segments. **(A)** Axial image at the level of the aortic root (Ao) showing the origins of the left and right coronary arteries (LCAo and RCAo), the left main coronary artery (LM), the left anterior descending artery (LAD), and the right coronary artery (RCA). **(B)** Oblique sagittal CPR image demonstrating the origin of the left coronary artery (LCAo) and the proximal, middle, and distal segments of the left anterior descending artery (LADp, LADm, and LADd). **(C)** Oblique coronal CPR image demonstrating the origin of the right coronary artery (RCAo) and the proximal, middle, and distal segments of the right coronary artery (RCAp, RCAm, and RCAd), as well as the left circumflex artery (LCX). **(D)** Oblique axial CPR image demonstrating the aortic root (Ao), the origins of the left and right coronary arteries (LCAo and RCAo), the left circumflex artery (LCX), and the right coronary artery (RCA). **(E)** Oblique axial CPR image demonstrating the aortic root (Ao), the origins of the left and right coronary arteries (LCAo and RCAo), the left anterior descending artery (LAD), and the right coronary artery (RCA). **(F)** Oblique axial CPR image demonstrating the left main coronary artery (LM), left anterior descending artery (LAD), and left circumflex artery (LCX).

Among all 125 patients, the overall visibility rate of the 11 coronary artery segments (score ≥ 3) was 90.4% (1,243/1,375), as summarized in Table 4. Visibility rates for the ostial, proximal, mid, and distal segments were 99.6% (249/250), 97.2% (486/500), 90.4% (226/250), and 75.2% (282/375), respectively. Visibility gradually decreased from proximal to distal segments; nevertheless, distal coronary segments remained assessable in approximately three-quarters of cases. High coronary artery visibility was maintained in both groups, with overall visibility rates of 91.4% (613/671) in the 80-kV group and 89.5% (630/704) in the 70-kV group. Visibility exceeded 80% for most coronary segments in both groups, although a modest reduction was observed in certain distal segments. No statistically significant differences in segmental visibility were identified between groups across all coronary segments (all *P* > 0.05) (Figure 2).

**Table 4:** Comparison of coronary artery visibility between the 80-kV and 70-kV groups.

Segment	Total segments evaluated, *n*	All patients (*n* = 125), *n* (%)		80-kV group (*n* = 61), *n* (%)		70-kV group (*n* = 64), *n* (%)		*P* value
		Score ≥3	Score <3	Score ≥3	Score <3	Score ≥3	Score <3	
Ostial segments	250	249 (99.6)	1 (0.4)	122 (100)	0 (0)	127 (99.2)	1 (0.8)	1.000
LCAo	125	125 (100)	0 (0)	61 (100)	0 (0)	64 (100)	0 (0)	1.000
RCAo	125	124 (99.2)	1 (0.8)	61 (100)	0 (0)	63 (98.4)	1 (1.6)	1.000
Proximal segments	500	486 (97.2)	14 (2.8)	237 (97.1)	7 (2.9)	249 (97.3)	7 (2.7)	0.927
LM	125	124 (99.2)	1 (0.8)	61 (100)	0 (0)	64 (100)	0 (0)	0.981
LADp	125	122 (97.6)	3 (2.4)	59 (96.7)	2 (3.3)	63 (98.4)	1 (1.6)	0.966
LCXp	125	116 (92.8)	9 (7.2)	57 (93.4)	4 (6.6)	59 (92.2)	5 (7.8)	1.000
RCAp	125	124 (99.2)	1 (0.8)	61 (100)	0 (0)	63 (98.4)	1 (1.6)	1.000
Middle segments	250	226 (90.4)	24 (9.6)	114 (93.4)	8 (6.6)	112 (87.5)	16 (12.5)	0.111
LADm	125	115 (92)	10 (8)	57 (93.4)	4 (6.6)	58 (90.6)	6 (9.4)	0.802
RCAm	125	111 (88.8)	14 (11.2)	57 (93.4)	4 (6.6)	54 (84.4)	10 (15.6)	0.108
Distal segments	375	282 (75.2)	93 (24.8)	140 (76.5)	43 (23.5)	142 (74)	50 (26)	0.568
LADd	125	95 (76)	30 (24)	48 (78.7)	13 (21.3)	47 (73.4)	17 (26.6)	0.492
LCXd	125	80 (64)	45 (36)	38 (62.3)	23 (37.7)	42 (65.6)	22 (34.4)	0.698
RCAd	125	107 (85.6)	18 (14.4)	54 (88.5)	7 (11.5)	53 (82.8)	11 (17.2)	0.306
All segments		1,243 (90.4)	132 (9.6)	613 (91.4)	58 (8.6)	630 (89.5)	74 (10.5)	0.240

Data are presented as number of segments (percentage). The total number of evaluated segments represents the number of coronary artery segments available for assessment in each corresponding segment category. Comparisons between groups were performed using the chi-square test. No statistically significant differences were observed between groups for any coronary segment.

### 3.3 Comparison of the objective evaluation of image quality

Overall, no significant differences were observed between the 70-kV and 80-kV groups in objective image quality parameters (Table 5). Image noise and vascular CT attenuation values did not differ significantly between groups (all *P* > 0.05). Similarly, signal-to-noise ratio (SNR) and contrast-to-noise ratio (CNR) values of the RCA, LAD, and LCX showed no significant intergroup differences (all *P* > 0.05), indicating that image quality in the 70-kV group was comparable to that in the 80-kV group.

**Table 5:** Comparison of objective image quality parameters between the 80-kV and 70-kV groups.

Parameter	80-kV group (*n* = 61)	70-kV group (*n* = 64)	*P* value
Image noise, HU	9.80 (5.35,11.95)	8.30 (6.05,11.42)	0.711
CT attenuation, HU			
RCA	415.58 ± 126.99	432.09 ± 192.70	0.671
LAD	340.50 (271.50,436.05)	366.80 (288.00,469.08)	0.440
LCX	316.40 (247.75,403.20)	291.2 (223.95,406)	0.360
SNR			
RCA	13.68 (9.79,18.32)	14.21 (9.65,19.98)	0.316
LAD	13.97 (10.35,19.62)	15.36 (11.73,23.06)	0.094
LCX	13.39 (9.82,16.99)	12.6 (8.89,18.29)	0.790
CNR			
RCA	62.20 (41.76,88.52)	69.52 (42.18,88.38)	0.621
LAD	50.74 (35.74,92.32)	57.41 (44.23,80.21)	0.340
LCX	51.34 (33.00,73.32)	50.75 (36.98,75.30)	0.906

SNR, signal-to-noise ratio; CNR, contrast-to-noise ratio; RCA, right coronary artery; LAD, left anterior descending artery; LCX, left circumflex artery. CT values of RCA are normally distributed and presented as mean ± standard deviation; other parameters are non-normally distributed and presented as median (interquartile range). Comparisons between groups were performed using independent-samples *t* test (normal) or Mann–Whitney *U* test (non-normal).

### 3.4 Comparison of radiation dose and iodine load

Significant differences were observed between groups in CTDIvol, DLP, and effective dose (ED) (all *P* < 0.001) (Table 6). The mean ED was 0.42 ± 0.06 mSv in the 80-kV group and 0.32 ± 0.05 mSv in the 70-kV group, corresponding to an approximate 24% reduction in radiation dose with the 70-kV protocol. No significant difference in iodine delivery was observed between groups (*P* = 0.752), with mean iodine delivery values of 1.35 ± 0.39 g I and 1.37 ± 0.32 g I in the 80-kV and 70-kV groups, respectively.

**Table 6:** Comparison of radiation dose and iodine load between the 80-kV and 70-kV groups.

Groups	CTDIvol (mGy)	DLP (mGy·cm)	ED (mSv)	Iodine delivery (g I)
80-kV group	4.30 ± 0.96	10.87 ± 1.46	0.42 ± 0.06	1.35 ± 0.39
70-kV group	5.15 ± 1.38	8.16 ± 1.25	0.32 ± 0.05	1.37 ± 0.32
In total	4.74 ± 1.26	9.48 ± 1.92	0.37 ± 0.07	1.36 ± 0.35

Data are presented as mean ± standard deviation. Statistical comparisons between groups were performed using the independent-samples *t* test.

## 4 Discussion

With advances in prospective ECG-triggering and iterative reconstruction techniques, cardiac CT has demonstrated good diagnostic performance in neonates and infants at acceptable radiation doses (4). However, most published neonatal and infant cardiac CT studies have still relied on 80-kV acquisition protocols, and evidence regarding further tube-voltage reduction in neonates remains limited (7, 10, 17).

Previous experimental studies using low tube voltage cardiac CT protocols have demonstrated that image quality can be maintained through optimization of acquisition parameters and reconstruction techniques, even under reduced radiation exposure conditions (18). Clinical studies using 70-kV CT protocols have further demonstrated the feasibility of low-kV CT angiography, particularly in pediatric populations, with preserved diagnostic image quality and reduced radiation dose (9). However, the application of 70-kV coronary CT angiography in neonates remains insufficiently investigated.

In the present study, we evaluated a 70-kV cardiac CT protocol with weight-adapted iodine delivery in a relatively large neonatal cohort (*n* = 64; mean age, 13.8 ± 9.6 days; mean body weight, 3.0 ± 0.8 kg). Compared with the conventional 80-kV protocol, the 70-kV protocol maintained coronary artery image quality and segmental visibility while achieving a 24% reduction in effective radiation dose. Importantly, satisfactory visualization was preserved even in distal coronary segments, supporting the feasibility of further dose optimization in neonatal cardiac CT.

Both subjective and objective image quality analyses demonstrated robust overall image quality of the 70-kV protocol. Interobserver agreement remained good to excellent across most coronary segments. Although *κ* values were numerically lower in several segments in the 70-kV group, *κ* statistics are primarily descriptive measures influenced by sample distribution and category prevalence; therefore, no formal statistical comparison between groups was performed (13, 15). Subjective image quality scores were comparable between groups in nearly all coronary segments, with significant differences observed only at the coronary ostia. However, both groups achieved near-maximal scores at these locations, suggesting limited clinical relevance and a possible ceiling effect of the 5-point scoring system (19). In addition, objective metrics, including CT attenuation, SNR, CNR, and image noise, did not significantly differ between protocols, further supporting the stability of image quality under ultra-low tube voltage (15, 20).

The preserved image quality at 70 kV is likely related to the physical characteristics of low-kV imaging. Lower tube voltage shifts photon energy closer to the K-edge of iodine, thereby enhancing intravascular attenuation and vascular contrast (21, 22). Although lower tube voltage generally increases image noise, the improved contrast enhancement may partially compensate for this effect, resulting in stable SNR and CNR. Previous studies have also shown that higher ASIR-V levels can help maintain image quality in pediatric low-dose CT imaging (22, 23). Nevertheless, objective image quality in the 70-kV group remained comparable rather than superior to that in the 80-kV group despite higher ASIR-V weighting, suggesting that the primary benefit of the low-kV protocol lies in maintaining acceptable image quality at reduced radiation exposure rather than relying solely on reconstruction-based noise suppression. The higher ASIR-V blending level used in the 70-kV group reflected the routine clinical protocol implemented at our institution for low-kV cardiac CT rather than an experimental modification intended to evaluate the independent effect of reconstruction strength.

Coronary artery visibility remained high across all segment levels in the present study, with an overall visibility rate exceeding 90% and no significant differences between groups. Notably, approximately three-quarters of distal coronary segments remained assessable despite ultra-low tube voltage acquisition. Because distal coronary visualization is generally considered one of the most challenging aspects of neonatal cardiac CT due to the small vessel caliber and increased susceptibility to motion artifacts (7), this finding may be clinically relevant. Previous pediatric cardiac CT studies using 80-kV protocols have demonstrated satisfactory visualization of proximal and mid coronary segments (7). Our findings further suggest that reducing tube voltage to 70 kV does not substantially compromise overall coronary assessability in neonates, particularly for distal coronary segments. This observation may be related to several neonatal anatomical and physiological characteristics, including small body size, shorter x-ray penetration paths, minimal perivascular fat, and the absence of coronary calcification, all of which may facilitate vascular conspicuity at lower photon energies (24).

Regarding radiation exposure, the 70-kV protocol achieved a 24% reduction in effective dose, with a mean effective dose of only 0.32 ± 0.05 mSv, which remains comparatively low relative to previously reported neonatal and infant cardiac CT studies using conventional 80-kV protocols (7, 17). Despite the lower effective dose, CTDIvol was relatively higher in the 70-kV group. This apparent discrepancy reflects the different dosimetric implications of these metrics. CTDIvol represents scanner radiation output per unit scan length and may increase because of automatic tube current modulation under low tube voltage conditions, whereas DLP and effective dose more accurately reflect cumulative patient radiation exposure (25). Consequently, the increased CTDIvol does not necessarily indicate a higher actual radiation burden. Although a formal figure-of-merit calculation was not performed, the combined assessment of radiation dose, objective and subjective image quality, and coronary artery visibility provided an integrated evaluation of protocol performance. This approach allowed assessment of the balance between dose reduction and image quality preservation, which is particularly relevant in neonatal cardiac CT where radiation exposure minimization is a primary consideration.

Optimization of iodine delivery also represents an important component of the dose optimization strategy. In this study, weight-adapted iodine delivery under low tube voltage conditions achieved adequate vascular enhancement without compromising image quality or visibility. This may reflect the synergistic relationship between lower photon energy and enhanced iodine attenuation efficiency, allowing stable vascular contrast despite reduced iodine exposure (21, 24). In addition, the relatively small circulating blood volume and rapid circulation in neonates may further improve contrast utilization efficiency (6). Overall, these findings support a balanced dose optimization strategy rather than pursuit of the lowest possible iodine dose alone.

Deep learning image reconstruction (DLIR) has recently shown promising noise-reduction capability in CT imaging. However, evidence regarding its application in neonatal cardiac CT remains limited, particularly under ultra-low-dose and high-heart-rate conditions (10, 26). In contrast, ASIR-V is an advanced model-based iterative reconstruction technique that has demonstrated effective noise reduction and image quality preservation in pediatric CT applications, including cardiac CT imaging (23, 27). Therefore, ASIR-V was selected in the present study because it represents a clinically established reconstruction approach with demonstrated stability in pediatric cardiac CT imaging.

## 5 Study limitations

This study has several limitations. First, this was a retrospective single-center study with a relatively small sample size, and patients were assigned according to sequential clinical imaging protocols rather than randomized allocation. Although consecutive patient inclusion and comparable baseline characteristics between groups may have reduced potential selection bias, the influence of temporal changes in clinical practice cannot be completely excluded. In addition, the sex distribution differed between groups, and the potential influence of sex on image quality or radiation exposure cannot be completely excluded. Second, this study primarily focused on coronary image quality and visibility rather than diagnostic accuracy for specific coronary anomalies. Third, different ASIR-V blending levels were applied between the two groups because this retrospective study reflected routine clinical practice rather than a prospective reconstruction optimization protocol. Therefore, the independent effects of tube voltage and reconstruction strength could not be completely separated. Future prospective studies with standardized reconstruction settings are warranted to further validate these findings.

## 6 Conclusions

In conclusion, cardiac angiography using a 70-kV protocol combined with weight-adapted iodine delivery is feasible in neonates and enables substantial radiation dose reduction while preserving coronary image quality and visibility. This dose-optimized approach provides a practical framework for balancing image quality, radiation exposure, and contrast administration in this highly vulnerable population. Further prospective and multicenter studies are warranted to validate these findings and to refine imaging protocols tailored to neonatal physiology.

## Data Availability

The original contributions presented in the study are included in the article/Supplementary Material, further inquiries can be directed to the corresponding author.
